# Investigation of Adhesive Resistance of Aluminum Alloy by Sandblasting and Electrochemical Machining

**DOI:** 10.3390/mi8030091

**Published:** 2017-03-17

**Authors:** Jianbing Meng, Xiaojuan Dong, Haian Zhou, Weihong Liu, Zhanmin Yin

**Affiliations:** School of Mechanical Engineering, Shandong University of Technology, Zibo 255000, China; dongxiaojuan@sdut.edu.cn (X.D.); zhouhaian1980@163.com (H.Z.); chnlwh@sdut.edu.cn (W.L.); yinzhanmin@sdut.edu.cn (Z.Y.)

**Keywords:** adhesive resistance, sandblasting, electrochemical machining, aluminum alloy

## Abstract

A novel method for fabricating an adhesive resistance surface is presented. Sandblasting and electrochemical machining were introduced to prepare micro-nano structures on the sample surface. Then, the prepared sample was immersed in a tridecafluoroctyltriethoxysilane ethanol solvent. The surface of the aluminum alloy sample roughened and covered with low-surface-energy chemical groups was examined by scanning electron microscope (SEM) and atomic force microscope (AFM). Surface wettability and adhesive resistance of the treated sample were characterized by water contact angles, area fraction, sliding angle and solid surface energy. Furthermore, the effects of some process parameters, such as sand size, current density, electrochemical machining time, and electrolyte concentration, on the contact angle, area fraction, sliding angle and the solid surface-energy of the modified sample surfaces were provided. The results show that the combination of binary micro-structures and surface modification of tridecafluoroctyltriethoxysilane plays a role to improve adhesive resistance of the aluminum alloy surface.

## 1. Introduction

Some soil animals, such as the dung beetle, earthworm and pangolin, have an interesting feature to prevent soils from entering their bodies [1,2,3]. This natural phenomenon has attracted great interest from researchers. They have attempted to prepare anti-adhesive surfaces on an aluminum alloy by various surface modification approaches. Jaszewski et al. investigated the adhesive resistance of two different ultra-thin Teflon-like films obtained by ion sputtering and plasma polymerization [4]. Their experimental results showed the interaction and degradation of adhesive resistance poly-tetrafluoroethylene (PTFE)-like films deposited on nickel shims in a hot embossing process. Sugihara and Enomoto developed a novel cutting tool with a banded micro-/nano-textured surface to improve the adhesive resistance and lubricity [5]. It was essential for achieving a good adhesive resistance in aluminum alloy cutting to improve fluid retention on the tool surface. Follmann et al. introduced an antibacterial agent (N-Trimethyl chitosan) and an anti-adhesive biopolymer into the modified polystyrene, in order to build multilayer films with adhesive resistance [6]. However, anti-adhesive coatings obtained from the above processes are likely to fall off, which results in anti-adhesive performance failure.

Creating binary structures exhibits a critical effect to improve anti-adhesive surfaces on aluminum and its alloys. A variety of approaches have been reported for fabricating the binary structures, including chemical etching, laser ablation, anodization, plasma electrolysis, electrodeposition, and so on. Wu et al. used electrochemical process to prepare nanowires with binary structures on aluminum alloys [7]. Li et al. fabricated a biomimetic-structural low-wettability surface with high stabilities and strong resistance on 2024 aluminum alloy [8]. First, ginkgo-leaf–like nanostructures were formed by a simple hydrothermal treatment. Then, a low-surface-energy compound was implemented to modify the wettability of 2024 aluminum alloy. Vengatesh et al. presented a simple method for preparing self-lubricating superhydrophobic hierarchical anodized aluminum oxide (AAO) surfaces by the electrochemical mild anodization of aluminum [9]. Liu et al. prepared the low-wettability surface on aluminum alloy via anodizing and polymeric coating [10]. They found that the micro-nano structures, combined with the low free energy, contributed to the superhydrophobicity of the aluminum alloy surfaces. Wu et al. prepared low-wettability aluminum alloy surfaces with modified nano-/micro-structures using different approaches, such as the acid etching method, the nitrate method, and the anodic oxidation method [11]. Huang et al. fabricated low-wettability thin films on aluminum alloy substrates by electrophoretic deposition process (EPD) using a stearic acid–functionalized zinc oxide nanoparticle suspension in alcohols at varying bath temperatures [12]. Li et al. formed layered double hydroxide films with different metal cations on anodized aluminum alloy [13]. Different types of metal cations showed great influence on the microstructures of the fabricated films. Although above techniques are effective to prepare micro-nano structures on aluminum alloy surfaces, some problems exist. For example, environmentally unfriendly materials, such as acid (hydrochloric acid, Nitric acid, etc.), and alkali liquids are usually used in chemical etching. Moreover, a complex process or an expensive apparatus limit the application of laser ablation and other methods.

In this paper, a combined method of sandblasting, electrochemical machining and surface modification with fluoroalkylsilane (FAS) is introduced to fabricate the binary microstructures and form a low-surface-energy polymer, which has an advantageous effect on enhancing the adhesive resistance of the aluminum alloy surface. Firstly, a microstructure on the sample surface is established by sandblasting; secondly, a nanostructure above the surface with the microstructure is fabricated by electrochemical machining; and finally, in order to form a low-surface-energy coating on the sample surface, the aforementioned sample is immersed in a tridecafluoroctyltriethoxysilane ethanol solvent. In this study, the surface morphology and surface wettability are investigated. Meanwhile, according to the analysis of the sliding angle and the adhesive energy, the surface adhesive resistance is revealed. The experimental results show that aluminum alloy surfaces with binary structures have an excellent adhesive resistance.

## 2. Materials and Methods

### 2.1. Materials

Aluminum alloys (2618) were purchased from Hanglong Metal Products Co., Ltd., (Foshan, China). A copper plate was obtained from CNMC Albetter Albronze Co., Ltd., (Liaocheng, China). Grained aluminum oxide (Al_2_O_3_) was used for sandblasting. Its type is 40, 60, 80, 100, 120 mesh (#), respectively. Their sizes are 425, 250, 180, 150 and 125 μm, respectively. FAS (tridecafluoroctyltriethoxysilane (C_8_F_13_H_4_Si(OCH_2_CH_3_)_3_) was supplied by Degussa Co., (Frankfurt, Germany), and the other experiment drugs, such as ethyl alcohol absolute, deionized water, and sodium phosphate (Na_3_PO_4_), purchased from Sinopharm Chemical Reagent Beijing Co., Ltd., (Beijing, China) was of analytical grade.

### 2.2. Fabrication of Adhesive Resistance on Aluminum Alloy

**Figure 1:**
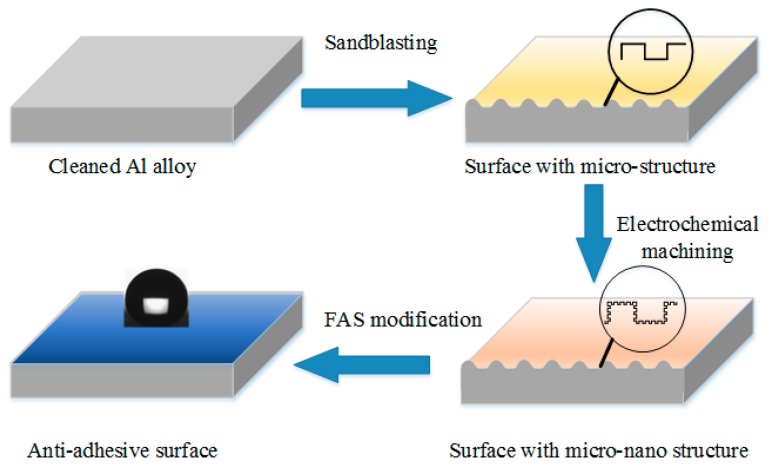
Schematic of the fabrication of adhesive resistance surfaces on aluminum alloys.

The procedures for fabrication of the adhesive resistance surfaces by the combined method of sandblasting and electrochemical machining were shown in Figure 1. A copper plate and an aluminum alloy plate, sizing of 30 mm × 30 mm × 2 mm, were used as the cathode and anode, respectively.

Before sandblasting, 1.0 wt % ethanol solution of fluoroalkylsilane was prepared by adding 1 g fluoroalkylsilane into 99 g anhydrous ethanol at room temperature in a beaker and stirring with a magnetic stirrer at a speed of 100 r/min for two hours. Aluminum alloy surfaces were polished by using metallographic emery papers. Then, these samples were treated by sandblasting using grained Al_2_O_3_. Sandblast equipment (ZS-1000R, Zhongshun Sand Blast Equipment Co., Ltd., Suzhou, China) was used for the aluminum alloy surface. The working pressure was 5 kg/cm^2^. In order to remove residual particles and other contaminants, ultrasonic cleaning method was used to clean sandblasted surfaces. After drying, the cleaned aluminum alloy plate and cathode plate were positioned in parallel. The distance between two inter-electrodes was 20 mm. They were treated by electrochemical machining in an aqueous solution of Na_3_PO_4_ at a direct current (DC) voltage of 50 V for 5–15 min at room temperature with a magnetic stirring at a speed of 500–1000 r/min. Then, with the used of deionized water, the obtained surfaces were again ultrasonically cleaned. Finally, an ethanol solution with 1.0 wt % FAS was applied to modify above cleaned surfaces of aluminum alloys. During the surface modification, ambient temperature and modification time were 80° and 5 h, respectively.

### 2.3. Characterization

The morphologies of aluminum surfaces were observed by a SIRION scanning electron microscope (SEM, Tescan, Shanghai, China) and a Multimode NS3a atomic force microscope (AFM, Veeco, Town of Oyster Bay, NY, USA), respectively. Contact angles and sliding angles were measured by a DSA100 optical contact angle meter (Kruss, Hamburg, Germany), using a droplet of water as an indicator. The average value of contact angles at three different positions of the sample surfaces was regarded as the final contact angle. The surface roughness was measured by a CLI2000 3D surface profiling (Taylor Hobson, Leicester, UK). The adhesive resistance was investigated by the sliding angle and the adhesive energy of aluminum alloy surfaces.

## 3. Results and Discussions

### 3.1. Surface Morphology and Wettability

**Figure 2:**
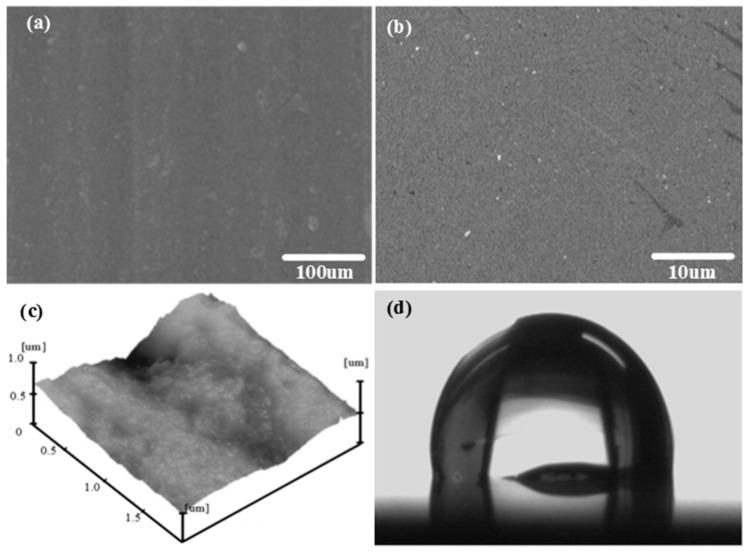
Images of scanning electron microscope (SEM), atomic force microscope (AFM) and a water droplet on the untreated aluminum alloy. (**a**) SEM image with 200 magnification; (**b**) SEM image with 2000 magnification; (**c**) AFM image; (**d**) water droplets with a volume of 5 μL.

**Figure 3:**
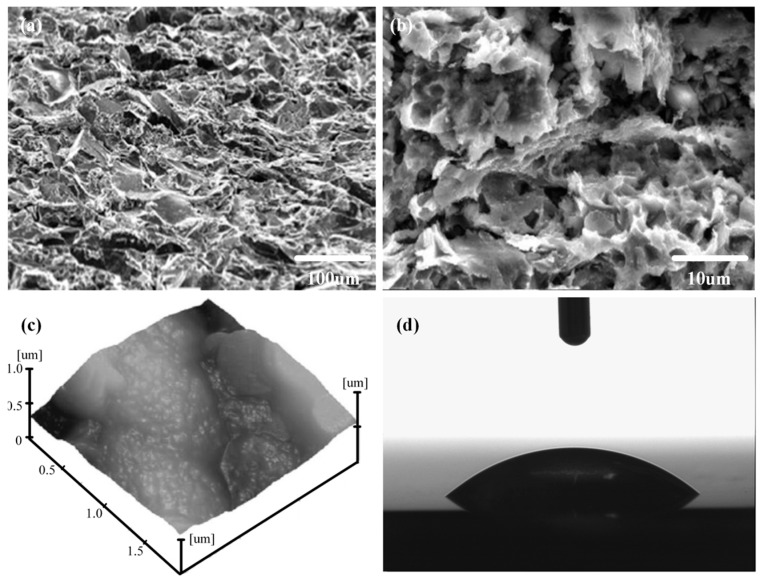
Images of SEM, AFM and a water droplet on the Al alloy surfaces after being treated by sandblasting. (**a**) SEM image with 200 magnification; (**b**) SEM image with 2000 magnification; (**c**) AFM image; (**d**) water droplets with a volume of 5 μL.

**Figure 4:**
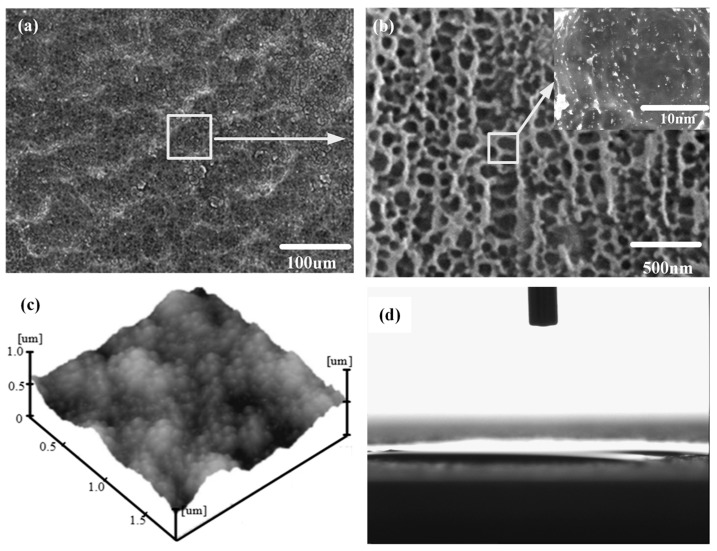
Images of SEM, AFM and a water droplet on the Al alloy surfaces after being treated by electrochemical machining. (**a**) SEM image with 200 magnification; (**b**) SEM image with 2000 and 50,000 magnification; (**c**) AFM image; (**d**) water droplets with a volume of 5 μL.

The original morphology of shot-free surfaces is shown in Figure 2. Before sandblasting, the sample surfaces exhibited a roughness value of 0.24 μm and a water contact angle (CA) value of 98.5°. Treated for 2 min by sandblasting with an 80 mesh sand size, the morphology of the aluminum alloy surface can be obtained from Figure 3. Figure 4 shows images of the SEM, AFM spectra of the sample surfaces, treated by electrochemical machining in 0.5 mol/L Na_3_PO_4_ aqueous solution with the application of 50 V voltage for 5 min, as well as the profile of one water droplet on the sample surface.

Figure 3a,b are the images with 200×, 2000× magnification, respectively. It can be seen that many pits with sizes of 10–40 µm are distributed on the aluminum alloy surface. The microstructure with the above pits was formed on the aluminum alloy surface by sandblasting. From Figure 4a,b, it can be found that many porous honeycombs with sizes of 50−250 nm were fabricated on the microstructure by electrochemical machining. Consequently, binary micro-nano structures are formed by the combination of sandblasting and electrochemical machining. Figure 3c and Figure 4c show the AFM images of the sample surface processed by sandblasting and electrochemical machining. In Figure 3c, the average roughness *Ra* is 0.48 μm on the roughened surface of the aluminum alloy sample after sandblasting and FAS modification. In Figure 4c, the average roughness *Ra* is 0.95 μm on the treated surface of the aluminum alloy sample after electrochemical machining.

In Figure 3b, besides micro-scale pits, a few nano-scale cavities existed in the sample surface treated by sandblasting. Compared with Figure 3b, Figure 4b shows a large number of nano-scale porous honeycombs dispersed uniformly on the whole surface fabricated by electrochemical machining. The roughness of the sample surface increased from 0.24 to 0.48 μm after sandblasting. As shown in Figure 4c, the roughness increased to 0.95 μm. After electrochemical machining, the aluminum surfaces exhibited superhydrophilic properties before fluorination, and water droplets spread on the surface completely with a contact angle of about 0°, as shown in Figure 4d. This phenomenon conforms to Wenzel’s theory that roughness enhances the hydrophilicity of hydrophilic surfaces. Micro-level protrusions and pits, as well as nano-level caves and mastoids in the pits, appeared to form the binary micro-nano structure of the sample surface.

From Figure 3c to Figure 4c, it can be seen that the average roughness of the latter is less than the former. This is because the Na_3_PO_4_ aqueous solution in this study is a neutral electrolyte. It is a non-linear electrolyte with a low current efficiency, which results in an oxygen evolution reaction occurring with the anode dissolution. Peaks of the rough microstructure are prone to being flatted by electrochemical machining. Consequently, the micro-nano structure with low roughness can be fabricated by sandblasting and electrochemical machining.

**Figure 5:**
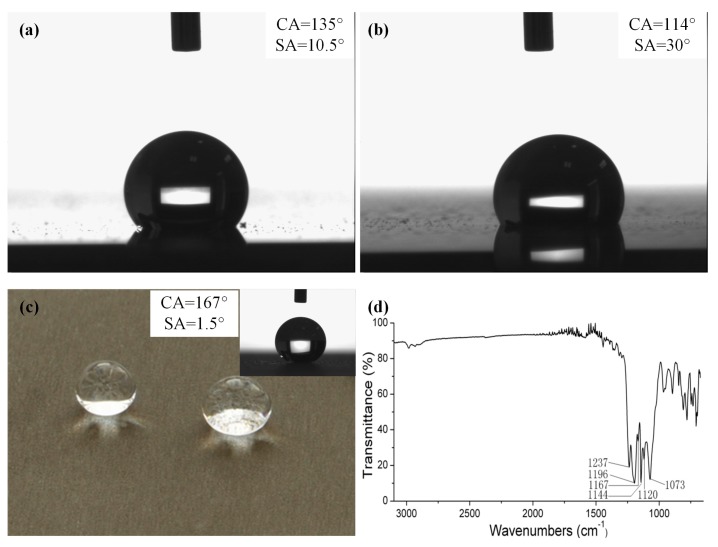
Images of contact angle (CA), sliding angle (SA) and Fourier transform infrared spectroscopy (FTIR) spectrum of the sample surface. (**a**) The treated surface after sandblasting and FAS modification; (**b**) the results after only electrochemical machining and FAS modification; (**c**,**d**) the results after sandblasting, electrochemical machining and FAS modification.

Figure 5 shows the characterization and wettability performance of the sample surface, treated by sandblasting, electrochemical machining and FAS modification. From Figure 5a, it can be observed that the water droplets can form a hemisphere (apparent contact angle of 135°) on the sample surface after treatment by sandblasting and FAS modification. However, with the secondary nanostructures (Figure 4b) being embedded on the surfaces of micro-scale caves (Figure 3b), the shape of the water droplets on the surfaces converts to a spherical shape instead (Figure 5c). After modification with FAS, compared with Figure 3, Figure 4 and Figure 5, the wettability changes from hydrophilicity to hydrophobicity. However, sliding angles (SA) in Figure 5a,b are much higher than the SAs in Figure 5c. As shown in Figure 5c, a water droplet cannot stably stick to the low-wettability surface. It rolls off immediately without any adhesion, indicating that the SA of the treated surface is as low as 1.5°. Hence, the addition of the secondary nanostructures and FAS modification play an important role in enhancing the low wettability and adhesive resistance.

Figure 5d shows the Fourier transform infrared spectroscopy (FTIR) spectra of the sample surfaces after the treatment by sandblasting, electrochemical machining and FAS modification. It can be seen that the absorption bands of 1073 cm^−1^ are assigned to Si–O–Si stretching, indicating that the aluminum surfaces have been uniformly covered by the fluoroalkylsilane polymer coating, not the monolayer. It can be seen that the intense absorption bands between 1237 and 1120 cm^−1^ are assigned to the C–F stretching of the –CF_3_ and –CF_2_ groups of the fluoroalkylsilane molecules, indicating that the low-surface-energy –CF_3_ and –CF_2_ groups have been successfully grafted onto the treated surface after modification with FAS.

### 3.2. Effect of Processing Parameters on Surface Wettablity

As shown in Figure 5c, a water droplet of 5 µL exhibits a typical spherical shape with a maximum contact angle of 167° and a tilting angle of 1.5° on the formed surface by sandblasting and electrochemical machining, followed by FAS modification. The low wettability of the fabricated surface on the aluminum alloy can be explained in terms of the Cassie-Baxter theory [14], which is described as follows:

(1) $$\cos\theta_{c}=f_{1}\cos\theta -f_{2}$$

where θ_c_ and θ are the contact angles on the rough and smooth surfaces, respectively; *f*_1_ and *f*_2_ (*f*_1_ + *f*_2_ = 1) are the area fractions of the solid surface *f*_1_ and trapped air in the voids among the micro-nano structures, respectively. This model indicates that the solid surface fraction is particularly important in determining the low wettability of the rough surface; *f*_1_ would decrease with the increase of the contact angle. According to the model, when a water droplet is placed on the formed surface by sandblasting, electrochemical machining and FAS modification, only about 3% serves as the contact area of the water droplet and the solid surface, but the remaining 97% serves as the contact area of the water droplet and air, respectively. This means that air pockets trapped between the solid and liquid can prevent direct contact between the water droplets and the aluminum alloy surface formed by sandblasting, electrochemical machining and FAS modification.

**Figure 6:**
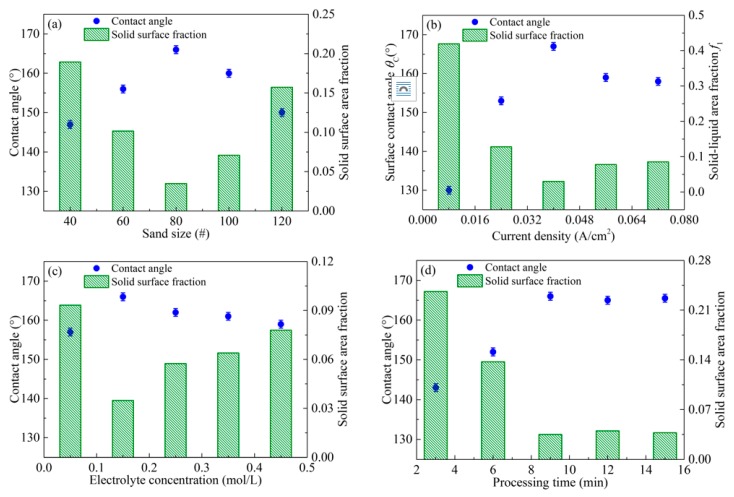
Influence of sand size (**a**), current density (**b**), electrolyte concentration (**c**) and processing time (**d**) on the contact angle and area fraction of the fabricated surface.

The variations of the contact angles and the area fraction of the solid surface *f*_1_ obtained on aluminum alloy surfaces by sandblasting and electrochemical machining, depending on the process parameters, such as sand size, processing current, electrolyte concentration, and processing time, are shown in Figure 6.

Figure 6a shows the variation of the contact angle and *f*_1_ with the sand size. The contact angle increases significantly with the decrease of the sand size from 40 to 80 mesh. It can reach 166° after the sample surfaces are treated by sandblasting with a sand size of 80#, electrochemical machining and FAS modification. The reason is that the rough microstructures become ever more uniform, and are prone to entrapping air to prevent direct contact between the treated surface and the water droplets. When the sand size is more than 80#, the contact angle decreases, because the less rough microstructure results in the increase of the fractional interfacial areas. Contrarily, the area fraction decreases to 0.035 with the increasing sand size from 40 to 80#, due to the well-distributed microstructures with proper roughness. As shown in Figure 6b, the contact angle increases and *f*_1_ decreases with the enhancement of the current density from 0.008 to 0.04 A/cm^2^. When the current density is more than 0.04 A/cm^2^, the contact angle decreases and *f*_1_ increases. The reason is that an excessive current density leads to the increase of the removal amount, and decreases the roughness of the binary micro-nano structures. Consequently, the increase of the contact area between the water droplets and the fabricated surface results in the decrease of the contact angle.

Figure 6c shows the variation of the contact angle and *f*_1_ with the Na_3_PO_4_ concentration. The increase of the electrolyte concentration leads to the improvement of the contact angles and the decrease of *f*_1_ until the concentration reaches 0.15 mol/L. It is because the conductivity and electrolytic corrosion increases with the electrolyte concentration. When the electrolyte concentration is greater than 0.15 mol/L, the interaction between the positive and negative ions increases, which results in the decline of the ion’s migration rate and the solution conductivity. Consequently, the contact angle and *f*_1_ are affected slightly by the Na_3_PO_4_ concentration in the range from 0.15 to 0.45 mol/L. As shown in Figure 6d, the contact angle increases and *f*_1_ decreases significantly with the processing time increasing from 3 to 9 min. The reason is that the oxide film obtained by electrochemical machining should be too thin to cover the original surface roughness. When the processing time is more than 9 min, the contact angle and *f*_1_ remain stable. This is because the grooves are completely covered by the oxide films, and the density and size of the pores increase with the anodic oxidation progress. Consequently, a large air fraction is generated on the formed surfaces with the fractal morphology.

### 3.3. Surface Anti-Adhesive Performance

Low adhesive forces are essential for improving the adhesive resistance of an aluminum alloy. However, it is difficult to directly measure the adhesive force of aluminum alloy surfaces. As is well known, anti-adhesive surfaces are dependent on the surface energy and the sliding angle [15,16]. In this paper, the sliding angle and solid surface energy are used to investigate the adhesive resistance of aluminum alloy surfaces prepared by sandblasting and electrochemical machining. The sliding angle can be measured by the DSA100 optical contact angle meter, and the solid surface energy can be calculated by the geometric mean method, which is written as:

(2) $$\gamma =\gamma_{S}^{L}+\gamma_{S}^{\mathrm{SP}}$$

(3) $$\gamma_{L}\left(1+{\cos\theta }_{c}\right)=2\sqrt{\gamma_{S}^{L}\gamma_{L}^{L}}+2\sqrt{\gamma_{S}^{\mathrm{SP}}\gamma_{L}^{\mathrm{SP}}}$$

where γ is the total surface-free energy, $\gamma_{S}^{L}$ is the London attraction of the van der Waals force, and $\gamma_{S}^{\mathrm{SP}}$ is the other type of non-dispersive component for physical interactions [17,18]. Further, $\gamma_{L}^{L}$ and $\gamma_{L}^{\mathrm{SP}}$ are the dispersive component and the specific component of liquid surface free energy (γ_L_) [19,20], respectively. The liquid used here is re-distillation water. The surface-free energy of the anti-adhesive surfaces on aluminum alloys can be obtained using Equations (2) and (3) with the measured contact angles.

**Figure 7:**
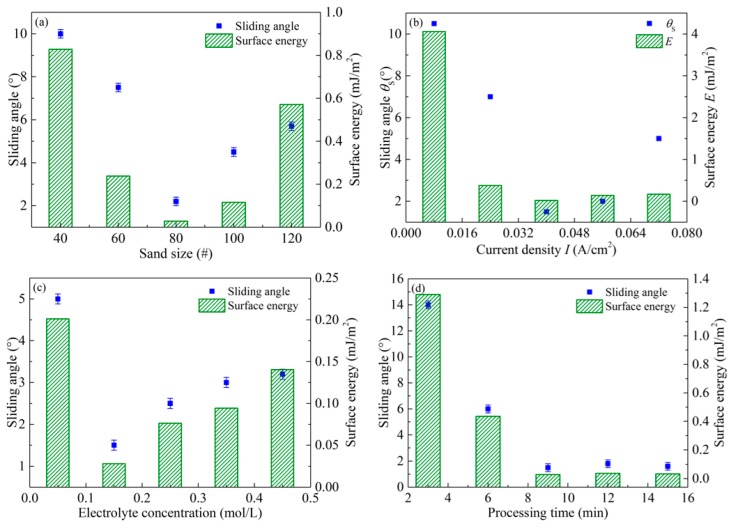
Influence of sand size (**a**), current density (**b**), electrolyte concentration (**c**) and processing time (**d**) on the sliding angle and the adhesive energy of the fabricated surface.

Their changes are presented in Figure 7.

From Figure 7a, it can be seen that surface energy first decreases with the decrement of the sand size. When the sand size is 80 mesh, the surface energy is lowest. With further decrement of the sand size, the surface energy enhances. This is due to binary structures entrapping air to reduce the contact area between the droplet and aluminum alloy surface. As shown in Figure 7b, the surface energy decreases with the increment of the current density, and it is down to 0.0209 mJ/m^2^ under the current density of 0.04 A/cm^2^. When the current density further increases, the surface energy increases instead. This reason is that the roughness of the fabricated surface is not high enough under the low current density, which is not in favor of the formation of steady air film reducing the adhesive force.

As shown in Figure 7c, the surface energy first decreases with the increase of the electrolyte concentration. When the Na_3_PO_4_ concentration is 0.15 mol/L, the surface energy is 0.02806 mJ/m^2^. Contrarily, the surface energy increases with the further increment of the electrolyte concentration. The reason is that there are few nano-level protrusions formed by electrochemical machining on the micro-level structure obtained from sandblasting, when the electrolyte concentration is less than 0.15 mol/L. From Figure 7d, it can be found that the surface energy first decreases with the increment of the processing time. When the time is 9 min, the surface energy decreases to 0.02806 mJ/m^2^. With the further increment of the processing time to 15 min, the surface energy is still 0.03277 mJ/m^2^. This is because the dynamic equilibrium between the dissolution of the anodized film and its formation is reached after 9 min during the processing of the aluminum alloy sample treated by electrochemical machining.

Furthermore, the changes of the sliding angle with different process parameters, such as sand size, current density, electrolyte concentration, and processing time, are also presented in Figure 6. The result is attributed to the combined effects of binary structures and chemical compositions. The former is a rough surface with micro-nano structures, which can trap air and reduce the contact between the aluminum alloy surfaces and liquid droplets. On the other hand, with low-surface-energy compounds, the rough surface is modified to render artificial adhesive resistance. During the modification, a polymerization reaction occurs between the surface groups and silanol groups, which can improve the adhesive resistance of the aluminum alloy surfaces.

## 4. Conclusions

The facile fabrication of the anti-adhesive surface on the aluminum alloy via sandblasting, electrochemical machining and FAS modification was investigated. The effects of sandblasting and electrochemical machining on the adhesive resistance of the formed surface were discussed. The results showed that an anti-adhesive surface with the binary micro-nano structures could be prepared by FAS modification after sandblasting and electrochemical machining. Furthermore, the anti-adhesive surface with binary structures and low-surface-energy compounds is affected by many process parameters, such as the sand size, current density, processing time and electrolyte concentration. As a result, to obtain anti-adhesive surfaces, it is essential to decrease the contact area of the solid/liquid interface and introduce some functional compounds with low surface energy.
